# Placental Epigenome-Wide Association Study Identified Loci Associated with Childhood Adiposity at 3 Years of Age

**DOI:** 10.3390/ijms21197201

**Published:** 2020-09-29

**Authors:** Valérie Gagné-Ouellet, Edith Breton, Kathrine Thibeault, Carol-Ann Fortin, Véronique Desgagné, Élise Girard Tremblay, Andres Cardenas, Renée Guérin, Patrice Perron, Marie-France Hivert, Luigi Bouchard

**Affiliations:** 1 Department of Biochemistry and Functional Genomics, Université de Sherbrooke, Sherbrooke, QC J1H 5N4, Canada; valerie.gagne.ouellet@usherbrooke.ca (V.G.-O.); edith.breton@usherbrooke.ca (E.B.); Kathrine.Thibeault@usherbrooke.ca (K.T.); Carol-Ann.Fortin@usherbrooke.ca (C.-A.F.); Veronique.desgagne@usherbrooke.ca (V.D.); elise_g_t@hotmail.com (É.G.T.); renee.guerin2@usherbrooke.ca (R.G.); 2 Department of Medical Biology, CIUSSS Saguenay-Lac-Saint-Jean—Hôpital Universitaire de Chicoutimi, Saguenay, QC G7H 5H6, Canada; 3 Division of Environmental Health Sciences, School of Public Health, University of California, Berkeley, CA 94720-7360, USA; andres.cardenas@berkeley.edu; 4 Department of Medicine, Université de Sherbrooke, Sherbrooke, QC J1H 5N4, Canada; Patrice.perron@usherbrooke.ca (P.P.); MHIVERT@PARTNERS.ORG (M.-F.H.); 5 Centre de Recherche du CHUS, Sherbrooke, QC J1H 5N4, Canada; 6 Diabetes Unit, Massachusetts General Hospital, Boston, MA 02114, USA; 7 Department of Population Medicine, Harvard Pilgrim Health Care Institute, Harvard Medical School, Boston, MA 02115, USA

**Keywords:** skinfolds thickness, epigenetics, fetal programming, DNA methylation, transcriptomics, EPIC array

## Abstract

The aim of this study was to identify placental DNA methylation (DNAm) variations associated with adiposity at 3 years of age. We quantified placental DNAm using the Infinium MethylationEPIC BeadChips. We assessed associations between DNAm at single-CpGs and skinfold thickness using robust linear regression models adjusted for gestational age, child’s sex, age at follow-up and cellular heterogeneity. We sought replication of DNAm association with child adiposity in an independent cohort. We quantified placental mRNA levels for annotated gene using qRT-PCR and tested for correlation with DNAm. Lower DNAm at cg22593959 and cg22436429 was associated with higher adiposity (*β* = −1.18, *q* = 0.002 and *β* = −0.82, *q* = 0.04). The cg22593959 is located in an intergenic region (chr7q31.3), whereas cg22436429 is within the *TFAP2E* gene (1p34.3). DNAm at cg22593959 and cg22436429 was correlated with mRNA levels at *FAM3C* (r_s_ = −0.279, *p* = 0.005) and *TFAP2E* (r_s_ = 0.216, *p* = 0.03). In an independent cohort, the association between placental DNAm at cg22593959 and childhood adiposity was of similar strength and direction (*β* = −3.8 ± 4.1, *p* = 0.36), yet non-significant. Four genomic regions were also associated with skinfold thickness within *FMN1*, *MAGI2*, *SKAP2* and *BMPR1B* genes. We identified placental epigenetic variations associated with adiposity at 3 years of age suggesting that childhood fat accretion patterns might be established during fetal life.

## 1. Introduction

Childhood obesity is now considered an overwhelming health problem as a consequence of a steadily rising prevalence over the last three decades [1]. In North America, as many as one-third of children are overweight or obese, and prevalence data are also concerning for developing countries [2,3]. Childhood obesity is associated with a wide range of complications (e.g., metabolic and cardiovascular dysfunctions) [4,5,6] with more than half of obese children at 5 years of age remaining obese in adulthood [7]. Therefore, unravelling its etiological factors and identifying early life biomarkers are essential steps to understand the pathophysiological processes leading to excess adiposity and improve childhood obesity prevention.

Recently, fat accretion and distribution patterns have emerged to be modulated by early-life determinants, suggesting that offspring’s metabolic health might be, at least partially, dependent on the first 1000 days of life [8]. Epigenetic modifications are key players in transcriptional activity regulation and might well contribute to the programming of growth and development in the offspring as well as to fat accretion in childhood. Epigenetic modifications are responsive to pre- and perinatal metabolic and environmental stress, as this critical period is characterized by rapid cellular divisions and differentiations combined with a strong DNA methyltransferase (DNMT) activity [9,10,11,12,13,14]. DNMTs catalyze the addition of a methyl group on the fifth carbon of a pyrimidine ring of a cytosine base resulting in DNA methylation (DNAm), the most studied epigenetic modification.

To date, a few studies have investigated the association between DNAm profile and childhood obesity using different tissues and phenotypes [15]. Accordingly, several single-site DNAm variations (i.e., epimutations or epivariations) have been associated with children’s body mass index (BMI), fat mass and skinfold thickness, but most of these were identified using a candidate gene approach and cross-sectional studies [16,17,18,19,20,21,22,23,24,25,26,27,28]. Although these studies may bring new insight into the research of childhood obesity’s biomarkers and pathogenesis, longitudinal studies using unbiased genome wide approaches are of critical importance to identify novel genes and pathways leading to obesity. The few published epigenome-wide association studies (EWAS) reporting epimutations (e.g., *KPRP*, *SCL9A10*, *MYLK2*) associated with measures of adiposity in children from 4 months to 8 years old were all performed in cord blood [23,28,29,30]. Nevertheless, considering its fetal origin and its sensitivity to in utero environmental variations in order to orchestrate materno-fetal nutrient exchanges, the placenta has a unique potential to reveal the early-life origins of childhood obesity. Accordingly, it has been recently shown that the placental transcriptome is associated with childhood obesity. Although the underlying biological mechanisms are yet to be unraveled [31].

In this study, we have used skinfold thickness as a surrogate of body fatness as it shows the best correlations with DXA fat mass measures (the gold standard) in children and is much less affected by lean mass than the BMI or the BMI z-score [32]. Therefore, the main goal of this study was to identify placental CpG sites and epigenomic regions associated with skinfold thickness to better define the fetal epigenetic determinants of childhood obesity.

## 2. Results

### 2.1. Participants Characteristics

Table 1 presents clinical data for the 262 mother–offspring dyads from Gen3G. Mothers were on average 28.6 ± 4.2 years old, with a mean BMI of 25.5 ± 5.5 kg/m^2^, and presented with mean glucose 1h-post 50-g glucose challenge test (GCT) of 5.0 ± 2.2 mmol/L at the first trimester of pregnancy (9.8 ± 2.3 weeks). At 24–28 weeks, women completed a 75 g oral glucose tolerance test (OGTT) and the mean 2 h glucose concentration was 5.9 ± 1.4 mmol/L. At delivery, mean gestational age of the offspring was 39.6 ± 1.0 weeks with a mean birthweight of 3.4 ± 0.4 kg. At the 3 years old research visit, on average, offspring were aged 40.5 ± 3.0 months and had a BMI of 16.1 ± 1.2 kg/m^2^, with a mean BMI z-score slightly higher (0.6 ± 1.1; *p* < 0.001) than their reference on the WHO growth chart (z-scores = 0). Mean ΣST was 17.5 ± 3.6 mm.

The clinical characteristics of the 187 participants from the 3D cohort included in the replication analysis are shown in Table S1. Briefly, the mothers were on average slightly older (31.4 ± 3.9 years old; *p* < 0.001) but leaner (BMI: 24.0 ± 4.5 kg/m^2^; *p* = 0.003) at first trimester of pregnancy when compared to Genetics of Glucose regulation in Gestation and Growth (Gen3G) mothers. Gestational age of the offspring at delivery (39.3 ± 1.2 weeks) was lower in comparison to Gen3G offspring (*p* = 0.02). Mean birthweight (3.4 ± 0.4 kg; *p* = 0.88) and early childhood ΣST (16.9 ± 4.9 mm; *p* = 0.25) were very similar to what we observed in Gen3G. However, 3D offspring were more than 1 year younger (25.8 ± 2.0 months; *p* < 0.001) at the follow-up visit than the Gen3G offspring.

### 2.2. Placental Epimutations Are Associated with Early-Childhood Adiposity in Gen3G Cohort

**Figure 1:**
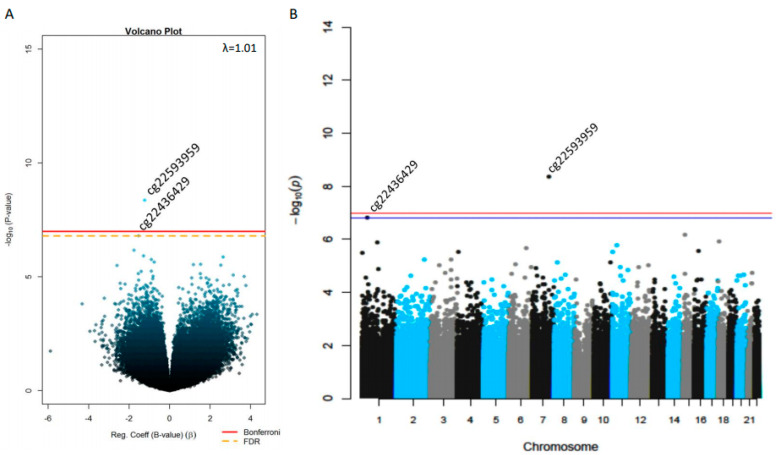
Epigenome-wide single-site associations between placental DNAm levels and childhood adiposity. (**A**) Volcano plot and (**B**) Manhattan plot shows the CpG-by-CpG analysis results of investigations between placental DNAm levels and children’s sum of skinfolds (subscapular + tricipital) in Gen3G participants. The False Discovery Rate (FDR) correction for multiple testing threshold (*q* < 0.05) is represented by a yellow doted-line in the Volcano plot and a blue line in the Manhattan plot, whilst the red line is the Bonferroni-corrected threshold (*p* < 1 × 10^−7^) in both graphs.

**Figure 2:**
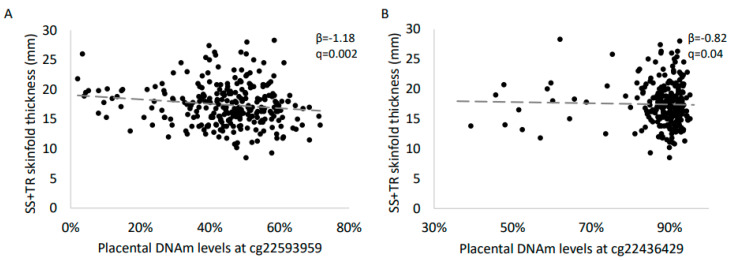
Association between DNAm levels at cg22593959 (**A**) and cg22436429 (**B**) and childhood adiposity. Robust linear regressions were computed in R with gestational age at birth, sex of the offspring, age at follow-up and cell composition estimation as covariates. Results were considered significant based on FDR correction for multiple testing (*q* < 0.05).

Results of the epigenome-wide association analysis between placental DNAm and ΣST are shown in Figure 1A. Across the epigenome, we detected one epimutation on chromosome 1 (cg22436429) and one on chromosome 7 (cg22593959) associated with ΣST at 3 years of age (False Discovery Rate, FDR, q < 0.05) (Figure 1B). Lower placental DNAm levels at both cg22436429 (*β* = −0.82 ± 0.21, *p* = 1.6 × 10^−7^, *q* = 0.04) and cg22593959 (*β* = − 1.18 ± 0.29, *p* = 4.4 × 10^−9^, *q* = 0.002) were associated with higher skinfold thickness at 3 years of age (Figure 2, Table 2).

Interestingly, both CpGs are within DNase hypersensitivity regions located at Chr1:36,042,645–36,043,855 and Chr7:121,184,805–121,185,215 (based on ENCODE data). When comparing the genomic structure, cg22436429 is in a CpG island within the *TFAP2E* gene (1p34.3) whereas the cg22593959 is located in an intergenic region near the 7q31.3 locus, nearby *CPDED1*, *WNT16* and *FAM3C* genes (Figures S1 and S2).

Regional analysis resulted in the identification of four additional loci associated with early-childhood adiposity (Table 3). The region with the strongest association (Sidak-adjusted *p* = 8.2 × 10^−6^) contains six CpG sites (cg19635897, cg19599407, cg15175581, cg09347959, cg24543970 and cg25310250) and is located at the *FMN1* gene locus on chromosome 15 (Figure S3). Differentially methylated regions (DMRs) within the *MAGI2* (*p* = 8.8 × 10^−6^) and *SKAP2* (*p* = 9.3 × 10^−5^) genes, which are both located on chromosome 7 (Figures S4 and S5), harbor seven (cg07448060, cg17463145, cg21784917, cg20996682, cg13382769, cg07985720 and cg24391460) and three CpG sites (cg20747577, cg11497410 and cg07473340), respectively. The last region (*p* = 2.6 × 10^−5^) within the *BMPR1B* (chromosome 4) had the highest number of CpG sites with nine (cg21066876, cg26878941, cg25288803, cg22273744, cg10549916, cg09771641, cg26603183, cg07341914 and cg22572902) (Figure S6). Lower DNAm levels at all four identified regions were associated with higher sums of skinfolds (Table S2).

### 2.3. Sex-Specific Effect of Fetal Programming of Adiposity

We further explored whether the identified epimutations and regions associated with early-childhood adiposity were influenced by sex. We did not observe significant interactions between sex and DNAm levels, yet at some identified regions, the associations seemed to be preferentially driven by one of the child sex strata (based on regression coefficients). Except for the region located on chromosome 4 (*BMPR1B*; position 95,972,466–95,972,790) where regression *β* coefficients were stronger in girls, the association with the three other regions (*FMN1*; chr 15: 33,360,195–33,360,337, *MAGI2*; chr 7: 79,083,753–79,084,166 and *SKAP2*; chr 7: 26,897,253–26,897,522) seemed to be driven by boys, although significant associations were observed with both sexes for most DMRs (Table S3).

### 2.4. Epigenetic Regulation of Gene Expression

**Figure 3:**
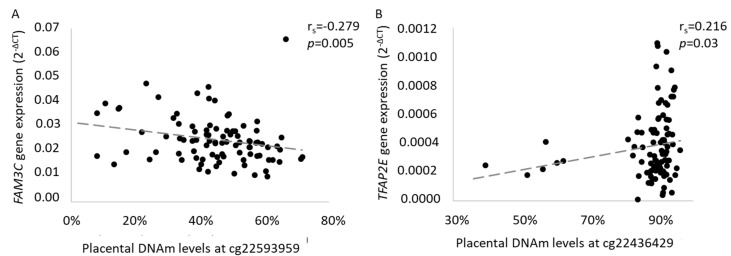
Associations between placental DNAm levels between (**A**) cg22593959 and *FAM3C* mRNA levels and (**B**) cg22436429 and *TFAP2E* mRNA levels. Spearman’s correlation tests were computed for each significant epimutations in the Gen3G cohort and mRNA levels of nearby or covering genes quantified relatively to the endogenous control *YWHAZ*.

Placental mRNA levels of genes located nearby the two epimutations identified (*WNT16*, *FAM3C*, *CPED1* and *TFAP2E*) and within the four regions (*FMN1*, *MAGI2*, *SKAP2* and *BMPR1B*) were quantified and tested for association with placental DNAm levels (Table 4) and offspring ΣST at 3 years of age. We first investigated the association between DNAm levels at cg22593959 and *FAM3C* and *CPED1* mRNA levels: *WNT16* mRNA was barely detectable and too weak to be further analyzed. We found that *FAM3C* DNAm was negatively correlated with its mRNA levels (r_s_ = −0.279, *p* = 0.005) (Figure 3A) whereas DNAm levels at cg22436429 were associated with lower *TFAP2E* gene levels (r_s_ = 0.216, *p* = 0.03) (Figure 3B). As outliers were previously removed with gaphunter, all data were kept in the analysis. We did not observe significant association between mRNA levels of *FAM3C* or *TFAP2E* and skinfold thickness (data not shown). None of the four regions identified by comb-p were associated with mRNA levels at their respective gene locus (Table 4).

### 2.5. Association between Maternal Characteristics and Placental Epimutations

We then tested the association between maternal anthropometric and metabolic profile and DNAm levels at both epimutations associated with ΣST. As shown in Table S4, placental DNAm levels at cg22593959 were not associated to maternal characteristics during pregnancy whereas cg22436429 was associated, although modestly, to the maternal blood lipid profile at the second trimester of pregnancy (low-density lipoprotein-cholesterol (LDL-C): *β* = 0.142, *p* = 0.03; total cholesterol: *β* = −0.128, *p* = 0.05).

### 2.6. Replication of CpG-by-CpG EWAS Results in 3D Birth Cohort

**Figure 4:**
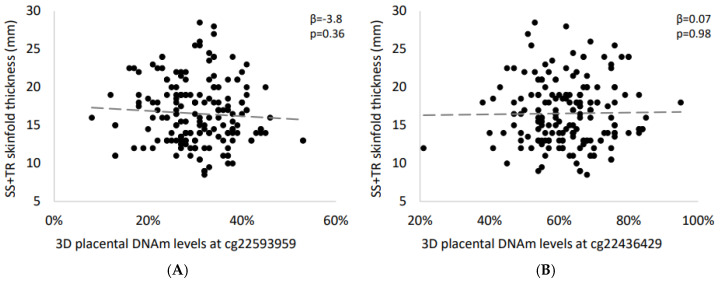
Association between placental DNAm levels at significant CpG sites and early-childhood adiposity in the independent 3D birth cohort. Spearman’s correlation tests were used to assess the association between skinfold thickness and DNAm levels at (**A**) cg22593959 and (**B**) cg22436429.

We sought to replicate our CpG-by-CpG significant sites (cg22593959 and cg22436429) in an independent birth cohort. Interestingly, the strength (based on *β* coefficients) and direction of the association between DNAm levels at cg22593959 and ΣST was similar in Gen3G (*β* = −3.8 ± 4.1 vs. −1.18 ± 0.29; Figure 4A), but not for cg22436429 (*β* = 0.07 ± 3.00 vs. −0.82 ± 0.21; Figure 4B). We also performed fine-mapping around the significant CpG sites. However, none of the other CpG sites tested nearby the cg22593959 nor within the *TFAP2E* gene locus were associated with childhood adiposity in 3D cohort (Table S5).

## 3. Discussion

In the current study, we identified two CpG sites and four regions at which placental DNAm levels were associated with children’s skinfold thickness [32,33]. Among the two CpGs, one was located within an intergenic region (locus 7q31.3) nearby the *WNT16-FAM3C*-*CPED1* locus, previously associated with metabolic syndrome [34], whereas the other was located within the *TFAP2E* gene locus. The two identified epimutations (i.e., cg22593959 and cg22436429) are likely regulating transcriptional activity of the *FAM3C* and *TFAP2E* genes, respectively, as supported by our expression analyses. Additionally, we identified four candidate epigenomic regions associated with skinfold thickness, which were located within *FMN1*, *MAGI2*, *SKAP2* and *BMPR1B* genes. Altogether, these results bring new supporting evidence that placental DNAm variation might predict fat accretion in early childhood.

Within the 7q31.3 locus, *FAM3C* seems the best potential target functionally related to the intergenic epimutation cg22593959. This is the only gene among the three tested showing correlation between the DNAm and mRNA levels. *FAM3C* is an ubiquitously expressed gene classified among the family with sequence similarity 3 (FAM3) gene family, which is involved in the regulation of hepatic glucose and lipid metabolism [35]. Although its specific role in energy regulation is still unclear, FAM3C overexpression in the liver of diabetic obese mice has been associated with improved glucose tolerance and insulin sensitivity [36]. Biologically, FAM3C upregulates Akt phosphorylation, which reduces neoglucogenesis and lipogenesis (and indirectly promotes lipolysis) [36]. FAM3C is also suspected to be involved in osteogenic cell differentiation [37] suggesting it might also have a role in adipogenesis. Indeed, osteogenesis and adipogenesis are closely related processes, especially since bone marrow-derived stromal cells (BMSCs) are multipotent cells that differentiate into lineages of mesenchymal tissues, such as chondrocytes, osteoblasts and adipocytes [38,39]. Together, these findings suggest that *FAM3C* epigenetic dysregulation could have shorter- and also longer-term impacts on the development of obesity. However, although placental *FAM3C* gene expression was not associated with early-childhood skinfold thickness in our study, it is possible that higher placental levels of *FAM3C* gene expression (associated to lower DNAm levels) may reflect *FAM3C* mRNA levels in other metabolically active tissues (e.g., adipose tissue) and therefore lead to greater adiposity. This will have to be assessed in further studies.

On the other hand, *TFAP2E* encodes a protein (AP-2E) in the transcriptional regulation by the AP-2 (TFAP2) family. This family includes transcription factors that bind to a DNA consensus sequence to activate several genes with biological functions in embryogenesis and growth of organs including eyes, face, skin and particularly central nervous system (CNS) [40,41]. The role of the central nervous system in food intake and energy homeostasis regulation is now well established [42]. In addition, as it was shown in mice and humans, this protein is highly suspected to orchestrate chondrogenesis and differentiation of muscle cells in embryos [43,44,45,46]. We herein reported that lower DNAm levels at cg22436429 were associated to lower *TFAP2E* gene expression in placenta and higher early-childhood adiposity. It is plausible that this gene might have protective effects against obesity. Hence, an epigenetic perturbation might lead to decreased rates of TFAP2E in embryos and might alter CNS or BMSCs normal development with further consequences on fat accretion. Interestingly, another member of the AP-2 family (*TFAP2B*) has been widely studied for its association with obesity and BMI variability [47,48,49,50,51], reinforcing that the AP-2 family has a crucial role to play in the development of obesity.

We also identified candidate placental epigenomic regions associated with early-childhood adiposity (i.e., *FMN1*, *MAGI2*, *SKAP2*, and *BMPR1B*), but we could not identify their functionally related target gene in the placenta, as DNAm levels within these regions were not associated with mRNA levels of the nearby genes. However, in light of our results, it is not possible to rule out that DNAm at these loci might be associated with their respective gene expression in other tissues and/or developmental windows. Still, whether through the regulation of BMSCs differentiation (*FMN1* and *BMPR1B*), glucose metabolism (*MAGI2*), or initiation of the inflammatory process (*SKAP2*), these genes could be relevant to the development of obesity [52,53,54,55].

### Strength and Limitations

Only a few studies assessed the longitudinal implication of the fetal methylome in the development of childhood excess fat accretion [56]. The use of a large number of full-term placentas from a longitudinal cohort is a strength of the study. Gen3G is a well-phenotyped prospective cohort: we recruited women at first trimester of pregnancy, and we followed mother–child pairs prospectively during childhood. We measured DNAm levels using the latest Illumina MethylationEPIC microarray, covering > 850,000 CpG sites across the human genome. Few currently published studies used the MethylationEPIC microarray and only one was performed to assess the association between leucocytes DNAm levels and obesity traits [56]. In addition, the similar direction of effect of the association between placental DNAm levels at cg22593959 and childhood total adiposity in an independent cohort supports the findings reported in the Gen3G cohort. However, the result of our replication did not reach statistical significance, which could be explained by the lower age at follow-up in 3D birth cohort, our incapacity to adjust the replication results for placental cell type heterogeneity, and/or differences in the sample collection procedure. Finally, the sample size might also have limited our ability to detect more modest effects of placental DNAm on childhood adiposity. EWAS (such as Genome-wide association studies, GWAS) results replication is challenging, needs time and performance in multiple cohorts before clear conclusion can be drawn. Accordingly, and even after considering the limitations reported above, our replication analyses should be considered as a strength especially as it supports in strengths and direction the association with cg22593959. Some other limitations are noteworthy to mention. Accordingly, although our study was conducted in Gen3G, a thoroughly phenotyped prospective birth cohort, we did not identify maternal factors potentially programming the epimutations reported in this study. Interestingly, maternal lipid profile (i.e., plasma LDL-C and total cholesterol concentrations) at the second trimester of pregnancy was modestly associated with DNAm levels at cg22436429 (within *TFAP2E*), but not with childhood adiposity. Moreover, the placental epimutations were not found to be correlated with maternal hyperglycemia as in a previous study from our group [57], suggesting that other maternal pregnancy factors may be responsible for programming these placental epigenetic disturbances [58,59]. Identified epivariations are likely to be either influenced by genetic factors or the consequences of fetal programming as they are untainted by post-natal exposures.

## 4. Materials and Methods

### 4.1. Genetics of Glucose regulation in Gestation and Growth (Gen3G) cohort Characteristics

Study participants were selected among the Genetics of Glucose regulation in Gestation and Growth (Gen3G) prospective birth cohort described previously [60]. Briefly, mothers were recruited during their first trimester of pregnancy. They fulfilled inclusion criteria if they were ≥18 years old, without pre-pregnancy diabetes and with a singleton pregnancy. Mother–child dyads were excluded if they encountered severe complication during pregnancy (e.g., high blood pressure or pre-eclampsia). A total of 262 participants were included in this study based on the availability of placental samples and if they had completed a 2 h post-75 g oral glucose tolerance test (OGTT; diagnosis test for gestational diabetes mellitus (GDM)) data between the 24th–28th weeks of pregnancy, gestational age ≥ 37 weeks of gestation at delivery and offspring’s indicators of adiposity at 3 years old. All mothers provided written consent at recruitment. This project was conducted in accordance with the Declaration of Helsinki and approved by the Centre Hospitalier Universitaire de Sherbrooke (CHUS) ethics committee (Project #2010-198, 07-027-A1, initially approved on September 2010 and renewed on 11th September 2020).

### 4.2. Replication: 3D Cohort Study (Design, Develop, Discover) Characteristics

For the replication study, 187 participants were randomly selected among the 3D Cohort Study (Design, Develop, Discover), a prospective pregnancy and birth cohort previously described [61,62]. Briefly, nine sites in Quebec recruited pregnant women and their partner during the first trimester of pregnancy and followed postnatally through 2 years after delivery. The replication study included 187 participants with available placenta samples, based on the following inclusion criteria: Caucasian with gestational age ≥ 37 weeks of gestation at delivery and adiposity measures in their child at 2 years of age. Participants with pre-gestational diabetes (Type 1 or 2) were excluded, alongside those with gestational hypertension or pre-eclampsia. All mothers gave written informed consent before enrollment in the study, in accordance with the Declaration of Helsinki.

### 4.3. Offspring’s Adiposity Measure in Gen3G and 3D Cohorts

In both cohorts, BMI was assessed and computed as z-scores based on the World Health Organization (WHO) growth chart reference for boys and girls. Subscapular (SS) and tricep (TR) thickness to the nearest 0.1 mm were measured by a Holtain skinfold caliper (Holtain Ltd., Crosswell, UK). The sum of SS and TR skinfolds (ΣST = SS + TR) was used as a proxy for overall adiposity [57,62] as it was previously associated with DNAm marks in cord blood [28]. Children from Gen3G with extreme values of BMI (n = 1; 33.6 kg/m^2^) or ΣST (n = 2; 8.5 and 48.5 mm) were excluded as they were >3 interquartile ranges (IQR) from the median.

### 4.4. Sample Collection and Nucleic Acids Extraction in Both Cohorts

Placental samples were collected after delivery (<30 min) by well-trained research staff. A total of 1 cm^3^ was collected on the fetal side, near the umbilical cord insertion (≤5 cm) and Gen3G samples were kept in RNAlater (Qiagen, Germantown, MD, USA). Samples from both cohorts were kept at −80 °C until DNA extraction. DNA and RNA were extracted using the All Prep DNA/RNA/Protein Mini Kit (Qiagen, Germantown, MD, USA) for both cohorts. Double strand DNA concentration were assessed using Quant-iT™ PicoGreen™ dsDNA Assay Kit (Qiagen, Germantown, MD, USA). Sodium-bisulfite conversions were performed prior to epigenome-wide methylation analyses following recommendations of the EpiTect Bisulfite Kit (Qiagen, Germantown, MD, USA). RNA concentrations and RNA integrity number (RIN) (mean 8.3 ± 0.9) were assessed with Agilent 2100 Bioanalyzer (Agilent RNA 6000 Nano Kit, Agilent Technologies, Santa Clara, CA, USA). Samples with RIN < 6 were excluded.

### 4.5. Infinium MethylationEPIC BeadChips in Gen3G Samples

In a previous study from our group [57], epigenome-wide DNAm levels were quantified in 448 placenta samples from the Gen3G placenta samples cohort using the Infinium MethylationEPIC BeadChip (Illumina, USA), which provides an improved coverage (i.e., 850 k CpG sites across the human genome) of enhancer and regulatory regions compared to the previous technologies [63,64].

Briefly, the samples were localized in random positions in both plates and chips to limit the impact of technical bias [57]. The preprocessing step performed on the overall samples included intra-sample quality controls (QCs) and probes clean-up steps using minfi library in R (Figure S7) [65]. Briefly, the QCs excluded samples that failed (n = 5) and with genotype (n = 6) and sex (n = 1) mismatches, alongside with technical duplicated (n = 10). From the original 448 placenta DNA samples from our previous study [57], 262 had offspring’s adiposity measures at 3 years old and were hence retained for the present study. First, data were processed using functional normalization with two principal components from control probes as a default [66]. The probe-type bias was then adjusted with Regressions on Correlated Probes (RCP), a method based on genomic vicinity to adjust type 2 probe distributions [67]. Detection *p*-values were computed and probes that did not reach significance for detection (*p* > 0.05) in 5% or more of the samples were excluded (n = 145 probes). Sex-chromosome-associated probes (n = 19,536), non-CpG probes (n = 2839) and SNP-associated probes (i.e., directly under the probe or at the single base extension at the CpG site (n = 83,222) and at CpG sites (n = 6075) with a minor allele frequency ≥ 5% were excluded from the analyses. The annotated probes suspected to be affected by cross-hybridization were also removed (n = 34,088) [63]. In addition, only probes with mean DNAm levels between 5 and 95% were retained for further analytical steps in the current analyses (n = 494,048) to avoid probes that show extreme levels of low or high DNAm [68]. Adjustment for potential technical bias was performed using the ComBat function from the sva package in R [69]. CpGs with DNAm level gaps >30% were identified (outliers) and removed using the gaphunter function (n = 5198 data points distributed among the 494 k probes) [70]. Prior to the analyses, the raw DNAm values were logit transformed into M-values to better fulfill regression modeling assumptions, although the effects were reported with the raw DNAm values to ease interpretation [71].

### 4.6. Gene Expression Analysis

To assess potential functionality in placenta of our top signals, RNA from a subset of 105 participants from the Gen3G cohort with both DNAm data and skinfold thickness measures at 3 years old was selected (RIN 8.3 ± 0.9). Prior to the amplification, complementary DNA (cDNA) has been generated with the High Capacity cDNA RT Kit (Thermofisher, Waltham, MA, USA). The quantitative real-time PCR (qRT-PCR) analyses were performed in duplicate using TaqMan^®^ Fast Universal PCR Master Mix (Thermofisher, Waltham, MA, USA). The mRNA levels were quantified for the genes located in the locus 7q31.3 (FAM3C: Hs02836787_g1, WNT16: Hs00365138_m1, CPED1: Hs00227735_m1), TFAP2E (Hs00698734_m1), FMN1 (Hs01396838_m1), MAGI2 (Hs00202321_m1), SKAP2 (Hs00919389_g1) and BMPR1B (Hs01010965_m1) (Thermofisher, Waltham, MA, USA) using the TaqMan^®^ Gene Expression Assays (Thermofisher, Waltham, MA, USA). The analyses were performed using the 7500 Real-Time PCR system (Thermofisher, Waltham, MA, USA). The qRT-PCRs were performed using 7500 Real-Time PCR system (Thermofisher, Waltham, MA, USA). The YWHAZ (Hs01122445_g1, Thermofisher, Waltham, MA, USA) was used as a reference gene as it was reported as stable in human placenta [72,73,74].

### 4.7. EpiTYPER Massarray in 3D Samples

The EpiTYPER Massarray (Sequenom^®,^ San Diego, CA, USA) approach was used to quantify DNAm levels in the placenta samples from the 3D Cohort at Genome Quebec. Briefly, this approach is based on the analysis of amplified bisulfite treated DNA followed by in vitro RNA transcription, uracile-specific cleavage and fragments analysis using MALDI-TOF mass spectrometry. The RNA fragments differ in size and mass depending of the methylation of the cytosines within the targeted sequence and the DNAm levels are determined using the EpiTYPER software. The PCR primers for the cg22436429 (and 30 more CpG in the vicinity) were: 5′-AGGTAGGTGGGTTTTTTGTTTTTT-3′ and 5′-ACTTTTCCAACCCAAATCTAAACC-3′, whereas the primers for the cg22593959 (and another CpG site nearby) were: 5′-AAGTGTGTGTTTGGTTTTGTTATAGG-3′ and 5′-AAAAACATCATATCATTAAAAATCCTC-3′. Five of the 187 DNA samples sent for replication analysis failed to amplify.

### 4.8. Statistical Analysis

The CpG-by-CpG analyses were all performed with R using individual robust linear regression models with placental DNAm on the M-value scale as the predictor and early-childhood adiposity represented by ΣST as the outcome in the Gen3G cohort. Robust linear regression was used to address potential heteroskedasticity [75]. Gestational age at delivery, sex of the offspring and its age at follow-up were included in the regression models as covariates. Analyses were also adjusted for cell composition heterogeneity using the first 5 principal components from ReFACTor, a reference-free method adjusting for cell-type heterogeneity adapted for all tissue, including placenta [76]. Inflation (λ) was assessed using quantile–quantile plots of *p*-values after adjustment for cellular heterogeneity (Figure S8). After adjustments, λ was 1.01. The reported results were all FDR-significant (*q*-value < 0.05). We also identified Bonferroni-significant probes (*p* < 1 × 10^−7^; 0.05/494 048 probes).

We further assessed epigenomic regions associated with early-childhood adiposity. To perform regional analysis, we used comb-p, a Python library, to determinate ΣST-associated differentially methylated regions (DMRs) in placenta [77]. Briefly, comb-p adjusts *p*-values from CpG-by-CpG regression models using the Stouffer–Liptak correction for highly autocorrelated CpGs within a 1000bp window and therefore combines the p-values. Combined p-values were adjusted for multiple testing with Sidak, which considers the number of potential regions created. DMRs were considered significant when *p* < 0.05. Regional association plots were computed with the R package comet.

The associations between DNAm levels and gene expression were investigated only for the placenta genes annotated or within 200 kb of the two FDR significant CpG sites. Normality was assessed using a Shapiro–Wilk distribution test and as both DNAm and mRNA levels were not distributed normally, even after log adjustment, we performed non-parametric tests. Spearman’s correlation tests were used to assess the statistical association between DNAm and mRNA levels. We also investigated the potential contribution of maternal anthropometric (e.g., pre-pregnancy BMI) and metabolic profile during pregnancy as we assessed the association between DNAm levels at significant epimutations and maternal variables using Spearman’s correlation tests.

As only epimutations from CpG-by-CpG were significantly associated with their respective gene expression, we only investigated these regions in the replication analysis. Linear regression models were used to investigate the association between significant placental epimutations and nearby CpG sites, and ΣST at 2 years old in DNA samples from 3D cohort. Replication and fine-mapping results were considered significant when *p* < 0.05. Spearman’s correlation and linear regression tests were performed on SPSS software (v25.0.0, IBM, USA, https://www.ibm.com/analytics/spss-statistics-software).

## 5. Conclusions

Our results suggest that placental DNAm levels measured at birth at specific loci are associated to early-childhood adiposity. The reported results support that the DNAm markers we identified in our CpG-by-CpG analyses might possibly induce a dysregulation of *FAM3C* and *TFAP2E* expression in placental tissues. These findings could provide new insights into the development of childhood obesity and provide potential new biomarkers for screening children at-risk of obesity as soon as at birth.

## Figures and Tables

**Table 1:** Characteristics of the mothers and children from Gen3G prospective cohort included in the analysis.

Participants’ Characteristics	Mean ± SD
**Maternal characteristics**	
1st trimester of pregnancy	
Age (years)	28.6 ± 4.2
BMI (kg/m^2^)	25.5 ± 5.5
Smoking during pregnancy	
Yes	7%
No	92%
Unknown	1%
Glucose 1h-post 50g-GCT (mmol/L)	5.0 ± 2.2
2nd trimester of pregnancy	
Fasting Glucose (mmol/L)	4.2 ± 0.3
Glucose 2h-post 75g-OGTT (mmol/L)	5.9 ± 1.4
Matsuda index	8.7 ± 5.3
HDL-cholesterol (mg/dL)	1.9 ± 0.4
LDL-cholesterol (mg/dL)	3.5 ± 0.9
Triglycerides (mg/dL)	1.8 ± 0.5
Total cholesterol (mg/dL)	6.2 ± 1.1
3rd trimester of pregnancy	
Gestational weight gain throughout pregnancy (kg)	11.4 ± 9.5
**Child characteristics**	
At birth	
Gestational age at birth (weeks)	39.6 ± 1.0
Sex (Boys/Girls)	
Boys	55%
Girls	45%
Birthweight (kg)	3.4 ± 0.4
At 3 years old	
Age (months)	40.5 ± 3.0
Weight (kg)	15.2 ± 1.7
BMI (kg/m^2^)	16.1 ± 1.2
Sum of skinfolds thicknesses (mm)	17.5 ± 3.6

**Table 2:** Adjusted difference in skinfold thickness in early childhood with a 1% change in placental DNA methylation levels.

CpG	Mean DNAm (SD)	Gene	Chromosome	Position	Gene Group	*β* Coefficient (SD)	*p* Value	*q* Value
cg22436429	88 (9)%	*TFAP2E*	Chr 1	36,043,084	Body	−0.82 (0.21)	1.6 × 10^−7^	0.04
cg22593959	45 (13)%		Chr 7	121,184,995		−1.18 (0.29)	4.4 × 10^−9^	0.002

**Table 3:** Regional placental DNA methylation associated to early-childhood adiposity.

Chromosome	Start Position	End Position	Minimal *p* Value	Nbr of CpGs	Sidak-Adjusted *p* Value	Gene	Direction of the Association
4	95,972,466	95,972,790	4.0 × 10^−4^	9	2.6 × 10^−5^	*BMPR1B*	*-*
7	26,897,253	26,897,522	2.0 × 10^−4^	3	9.3 × 10^−5^	*SKAP2*	*-*
7	79,083,753	79,084,166	1.2 × 10^−5^	7	8.8 × 10^−6^	*MAGI2*	*-*
15	33,360,195	33,360,337	2.0 × 10^−4^	6	8.2 × 10^−6^	*FMN1*	*-*

**Table 4:** Association between DNAm levels at both single-site and epigenomic regions, and transcriptional activity.

cg or Epigenomic Region	Gene	Correlation
cg22593959	*CPED1*	r_s_ = −0.111 *p* = 0.28
	*WNT16*	Not detected
	*FAM3C*	r_s_ = −0.279 *p* = 0.005
cg22436429	*TFAP2E*	r_s_ = 0.216 *p* = 0.03
*FMN1*	*FMN1*	r_s_ = −0.004 *p* = 0.97
*MAGI2*	*MAGI2*	r_s_ = 0.038 *p* = 0.71
*SKAP2*	*SKAP2*	r_s_ = 0.129 *p* = 0.20
*BMPR1B*	*BMPR1B*	r_s_ = 0.068 *p* = 0.51

## Supplementary Materials

Supplementary Materials can be found at https://www.mdpi.com/1422-0067/21/19/7201/s1. Figure S1: Schematic representation of the preprocessing steps including probes quality controls and clean-up, Figure S2: QQ-plot for the association between placental methylome and childhood adiposity, Figure S3: Regional association plot and correlation heatmap of CpGs located near cg22593959 from the CpG-by-CpG analysis, Figure S4: Regional plot and correlation heatmap of CpGs located near cg22436429 from the CpG-by-CpG analysis, Figure S5: Regional plot and correlation heatmap of CpGs located in the *FMN1* gene locus from the regional analysis, Figure S6: Regional plot and correlation heatmap of CpGs located in the MAGI2 gene locus from the regional analysis, Figure S7: Regional plot and correlation heatmap of CpGs located in the *SKAP2* gene locus from the regional analysis, Figure S8: Regional plot and correlation heatmap of CpGs located in the *BMPR1B* gene locus from the regional analysis, Table S1: Characteristics of the mothers and children from 3D prospective cohort and comparison with Gen3G, Table S2: Detailed EWAS results for the epigenomic regions associated with early-childhood adiposity in the Gen3G cohort, Table S3: Sex-stratification of associations between placental DNAm levels at epimutation sites and early-childhood adiposity in the Gen3G cohort, Table S4: Associations between placental DNA methylation levels in significant CpG sites and anthropometric and metabolic profile of mothers from the Gen3G birth cohort, Table S5: Associations between placental DNA methylation levels in fine-mapped CpG sites and sum of skinfold thickness in children at 2 years of age from the 3D birth cohort.

## Abbreviations

BMI-z	body mass index z-scores
BMPR1B	bone morphogenetic protein receptor type 1B
BMSC	bone marrow-derived stromal cells
CPED1	cadherin-like and PC-esterase domain containing 1
CNS	central nervous system
DMR	differentially methylated region
DNAm	DNA methylation
DNMT	DNA methyltransferase
EWAS	epigenome-wide association study
FAM3C	family with sequence similarity 3 member C
FMN1	formin 1
GCT	glucose challenge test
GDM	gestational diabetes mellitus
Gen3G	Genetics of Glucose regulation in Gestation and Growth
HDL-C	high-density lipoprotein cholesterol
IQR	interquartile range
LDL-C	low-density lipoprotein cholesterol
MAGI2	membrane-associated guanylate kinase
QC	quality control
RIN	RNA integrity number
SKAP2	Src kinase-associated phosphoprotein 2
SS	suprascapular
ST	skinfold thickness
TFAP2E	transcription factor AP-2 epsilon
TR	tricipital
WHO	World Health Organization
WNT16	Wnt family member 16
