# Supplementary material for: Placental Epigenome-Wide Association Study Identified Loci Associated with Childhood Adiposity at 3 Years of Age
Source: Int J Mol Sci. 2020 Sep 29;21(19):7201. doi: 10.3390/ijms21197201 (PMC7582906; doi:10.3390/ijms21197201)
Supplement: Supplementary file 1 [file ijms-21-07201-s001.zip › Table S1.docx]

Table S1. Characteristics of the mothers and children from 3D prospective cohort and comparison with Gen3G

| **Participant clinical data n=187** | **Mean ± SD** | **Comparison with Gen3G (p value)** |
| --- | --- | --- |
| **Maternal characteristics** |  |  |
| **1^st^ trimester of pregnancy** |  |  |
| Age (years) | 31.4 ± 3.9 | **<0.001** |
| BMI (kg/m^2^) | 24.0 ± 4.5 | **0.003** |
| Smoking during pregnancy |  | **<0.001** |
| Yes | 10% |  |
| No | 89% |  |
| Unknown | 1% |  |
| **Child characteristics** |  |  |
| **At birth** |  |  |
| Gestational age at birth (weeks) | 39.3 ± 1.2 | **0.02** |
| Sex (Boys/Girls) |  | 0.17 |
| Boys | 47% |  |
| Girls | 53% |  |
| Birthweight (kg) | 3.4 ± 0.4 | 0.88 |
| **At 2 years old** |  |  |
| Age (months) | 25.8 ± 2.0 | **<0.001** |
| Weight (kg) | 17.6 ± 12.3 | **0.02** |
| BMI (kg/m^2^) | 22.7 ± 15.3 | **<0.001** |
| Sum of skinfolds thicknesses (mm) | 16.9 ± 4.9 | 0.25 |

Comparison between Gen3G and 3D clinical characteristics of the participants were performed using Student T test, or Chi-square for categorial variables (i.e., sex of the child and smoking during pregnancy).
