# Supplementary material for: Placental Epigenome-Wide Association Study Identified Loci Associated with Childhood Adiposity at 3 Years of Age
Source: Int J Mol Sci. 2020 Sep 29;21(19):7201. doi: 10.3390/ijms21197201 (PMC7582906; doi:10.3390/ijms21197201)
Supplement: Supplementary file 1 [file ijms-21-07201-s001.zip › Table S2.docx]

| probeID | BETA | SE | P_VAL | N | UCSC_RefGene_Name | chr | pos | Relation_to_Island | Probe_rs | Probe_maf |
| --- | --- | --- | --- | --- | --- | --- | --- | --- | --- | --- |
| cg19635897 | -4.144 | 0.144 | 0.259 | 274 | FMN1 | chr15 | 33360195 | OpenSea | NA | NA |
| cg19599407 | -2.578 | 0.150 | 0.267 | 274 | FMN1 | chr15 | 33360214 | OpenSea | NA | NA |
| cg15175581 | -4.973 | 0.137 | 0.119 | 274 | FMN1 | chr15 | 33360262 | OpenSea | NA | NA |
| cg09347959 | -2.416 | 0.154 | 0.409 | 274 | FMN1 | chr15 | 33360271 | OpenSea | NA | NA |
| cg24543970 | -4.219 | 0.104 | 0.062 | 274 | FMN1 | chr15 | 33360329 | OpenSea | NA | NA |
| cg25310250 | -2.338 | 0.172 | 0.197 | 274 | FMN1 | chr15 | 33360337 | OpenSea | NA | NA |
| cg07448060 | -1,110 | 0.034 | 0.005 | 274 | MAGI2 | chr7 | 79083753 | Island | NA | NA |
| cg17463145 | -2,153 | 0.118 | 0.005 | 274 | MAGI2 | chr7 | 79084011 | S_Shore | NA | NA |
| cg21784917 | -1,139 | 0.098 | 0.096 | 274 | MAGI2 | chr7 | 79084038 | S_Shore | NA | NA |
| cg20996682 | -1,516 | 0.097 | 0.009 | 274 | MAGI2 | chr7 | 79084048 | S_Shore | NA | NA |
| cg13382769 | -1,254 | 0.079 | 0.009 | 274 | MAGI2 | chr7 | 79084078 | S_Shore | rs114359711 | 0.016 |
| cg07985720 | -1,466 | 0.087 | 0.008 | 274 | MAGI2 | chr7 | 79084127 | S_Shore | NA | NA |
| cg24391460 | -0,958 | 0.083 | 0.127 | 274 | MAGI2 | chr7 | 79084166 | S_Shore | rs114359711 | 0.016 |
| cg20747577 | -0,732 | 0.043 | 0.115 | 274 | SKAP2 | chr7 | 26897253 | OpenSea | NA | NA |
| cg11497410 | -0,724 | 0.049 | 0.092 | 274 | SKAP2 | chr7 | 26897311 | OpenSea | NA | NA |
| cg07473340 | -0,804 | 0.059 | 0.046 | 274 | SKAP2 | chr7 | 26897522 | OpenSea | NA | NA |
| cg21066876 | -1,052 | 0.093 | 0.064 | 274 | BMPR1B | chr4 | 95972466 | OpenSea | NA | NA |
| cg26878941 | -0,469 | 0.080 | 0.296 | 274 | BMPR1B | chr4 | 95972508 | OpenSea | NA | NA |
| cg25288803 | -1,305 | 0.066 | 0.155 | 274 | BMPR1B | chr4 | 95972539 | OpenSea | NA | NA |
| cg22273744 | -1,480 | 0.091 | 0.014 | 274 | BMPR1B | chr4 | 95972614 | OpenSea | NA | NA |
| cg10549916 | -1,689 | 0.092 | 0.017 | 274 | BMPR1B | chr4 | 95972631 | OpenSea | NA | NA |
| cg09771641 | -2,786 | 0.134 | 0.015 | 274 | BMPR1B | chr4 | 95972652 | OpenSea | NA | NA |
| cg26603183 | -1,676 | 0.085 | 0.001 | 274 | BMPR1B | chr4 | 95972744 | OpenSea | NA | NA |
| cg07341914 | -2,360 | 0.132 | 0.002 | 274 | BMPR1B | chr4 | 95972775 | OpenSea | NA | NA |
| cg22572902 | -2,434 | 0.131 | 0.003 | 274 | BMPR1B | chr4 | 95972790 | OpenSea | NA | NA |

Table S2. Detailed EWAS results for the epigenomic regions associated with early-childhood adiposity in the Gen3G cohort.
