# Supplementary material for: Placental Epigenome-Wide Association Study Identified Loci Associated with Childhood Adiposity at 3 Years of Age
Source: Int J Mol Sci. 2020 Sep 29;21(19):7201. doi: 10.3390/ijms21197201 (PMC7582906; doi:10.3390/ijms21197201)
Supplement: Supplementary file 1 [file ijms-21-07201-s001.zip › Table S3.docx]

**Table S3.** Sex-stratification of associations between placental DNAm levels at epimutation sites and early-childhood adiposity in the Gen3G cohort.

|  |  | **Boys (n=144)** | | | **Girls (n=118)** | | |  |
| --- | --- | --- | --- | --- | --- | --- | --- | --- |
| **Gene** | **probe ID** | **β coefficient** | **SE** | **p value** | **β coefficient** | **SE** | **p value** | **Interaction p value** |
| none | cg22593959 | -1.16 | 0.29 | <0.001 | -1.19 | 0.29 | <0.001 | 0.264 |
| *TFAP2E* | cg22436429 | -1.73 | 0.45 | <0.001 | -1.61 | 0.27 | <0.001 | 0.012 |
| *FMN1* | cg19635897 | -1.15 | 0.77 | 0.136 | -0.45 | 0.77 | 0.556 | 0.334 |
| *FMN1* | cg19599407 | -2.54 | 0.73 | <0.001 | -1.76 | 0.79 | 0.026 | 0.570 |
| *FMN1* | cg15175581 | -2.32 | 0.48 | <0.001 | -1.27 | 0.56 | 0.024 | 0.334 |
| *FMN1* | cg09347959 | -3.17 | 0.72 | <0.001 | -1.89 | 0.96 | 0.048 | 0.570 |
| *FMN1* | cg24543970 | -1.98 | 0.67 | 0.003 | -0.97 | 0.60 | 0.108 | 0.310 |
| *FMN1* | cg25310250 | -2.94 | 0.82 | <0.001 | -1.41 | 0.86 | 0.102 | 0.638 |
| *MAGI2* | cg07448060 | -0.60 | 0.19 | 0.002 | -0.31 | 0.19 | 0.101 | 0.672 |
| *MAGI2* | cg17463145 | -1.59 | 0.55 | 0.004 | -1.13 | 0.67 | 0.094 | 0.805 |
| *MAGI2* | cg21784917 | -1.17 | 0.48 | 0.014 | -0.94 | 0.53 | 0.078 | 0.608 |
| *MAGI2* | cg20996682 | -1.64 | 0.47 | 0.001 | -0.93 | 0.55 | 0.090 | 0.195 |
| *MAGI2* | cg13382769 | -1.42 | 0.43 | 0.001 | -0.99 | 0.39 | 0.010 | 0.207 |
| *MAGI2* | cg07985720 | -1.56 | 0.53 | 0.003 | -0.92 | 0.44 | 0.036 | 0.516 |
| *MAGI2* | cg24391460 | -0.98 | 0.62 | 0.115 | -0.76 | 0.34 | 0.026 | 0.608 |
| *SKAP2* | cg20747577 | -0.83 | 0.22 | <0.001 | -0.21 | 0.33 | 0.516 | 0.090 |
| *SKAP2* | cg11497410 | -0.90 | 0.20 | <0.001 | -0.18 | 0.33 | 0.577 | 0.981 |
| *SKAP2* | cg07473340 | -1.16 | 0.21 | <0.001 | -0.22 | 0.45 | 0.622 | 0.484 |
| *BMPR1B* | cg21066876 | -0.67 | 0.54 | 0.212 | -1.53 | 0.59 | 0.009 | 0.986 |
| *BMPR1B* | cg26878941 | -0.79 | 0.52 | 0.133 | -1.02 | 0.48 | 0.033 | 0.109 |
| *BMPR1B* | cg25288803 | -0.32 | 0.37 | 0.392 | -0.91 | 0.36 | 0.011 | 0.794 |
| *BMPR1B* | cg22273744 | -0.87 | 0.38 | 0.022 | -1.44 | 0.56 | 0.010 | 0.287 |
| *BMPR1B* | cg10549916 | -0.60 | 0.46 | 0.188 | -1.41 | 0.50 | 0.005 | 0.872 |
| *BMPR1B* | cg09771641 | -1.13 | 0.79 | 0.150 | -2.91 | 0.69 | <0.001 | 0.283 |
| *BMPR1B* | cg26603183 | -1.12 | 0.46 | 0.014 | -1.71 | 0.56 | 0.002 | 0.559 |
| *BMPR1B* | cg07341914 | -1.34 | 0.61 | 0.028 | -2.59 | 0.75 | 0.001 | 0.698 |
| *BMPR1B* | cg22572902 | -1.30 | 0.64 | 0.043 | -2.48 | 0.67 | <0.001 | 0.520 |
|  |  |  |  |  |  |  |  |  |
