# Supplementary material for: Placental Epigenome-Wide Association Study Identified Loci Associated with Childhood Adiposity at 3 Years of Age
Source: Int J Mol Sci. 2020 Sep 29;21(19):7201. doi: 10.3390/ijms21197201 (PMC7582906; doi:10.3390/ijms21197201)
Supplement: Supplementary file 1 [file ijms-21-07201-s001.zip › Table S4.docx]

Table S4. Associations between placental DNA methylation levels in significant CpG sites and anthropometric and metabolic profile of mothers from the Gen3G birth cohort.

| probe ID | BMI at 1^st^ trimester of pregnancy | Fasting glucose at 2^nd^ trimester of pregnancy | 2h-post 75g-OGTT | Matsuda index | HOMA-IR at 2nd trimester of pregnancy | Gestational weight gain throughout pregnancy | HDL-C at 2^nd^ trimester of pregnancy | LDL-C at 2^nd^ trimester of pregnancy | TG at 2^nd^ trimester of pregnancy | Total Cholesterol at 2^nd^ trimester of pregnancy |
| --- | --- | --- | --- | --- | --- | --- | --- | --- | --- | --- |
| cg22593959 | β=-0.001 p=0.490 | β=0.003 p=0.881 | β=0,003 p=0.609 | β=0.001 p=0.737 | β=0.001 p=0.930 | β=-0.001 p=0.436 | β=-0.039 p=0.724 | β=-0.054 p=0.633 | β=-0.018 p=0.726 | β=0.058 p=0.609 |
| cg22436429 | β=-0.001 p=0.853 | β=0.011 p=0.356 | β=0,003 p=0.373 | β=-0.001 p=0.132 | β=0.001 p=0.001 | β=-0.001 p=0.806 | β=0.117 p=0.066 | **β=0.142 p=0.029** | β=0.044 p=0.140 | **β=-0.128 p=0.048** |

Associations between maternal profile and DNA methylation levels at significant epimutations were assessed using linear regression models with gestational age at delivery, sex of the offspring, maternal age and placental cellular heterogeneity (5 PCs) as covariates.
