# Supplementary material for: Placental Epigenome-Wide Association Study Identified Loci Associated with Childhood Adiposity at 3 Years of Age
Source: Int J Mol Sci. 2020 Sep 29;21(19):7201. doi: 10.3390/ijms21197201 (PMC7582906; doi:10.3390/ijms21197201)
Supplement: Supplementary file 1 [file ijms-21-07201-s001.zip › Table S5.docx]

**Table S5.** Associations between placental DNA methylation levels in fine-mapped CpG sites and sum of skinfold thickness in children at 2 years of age from the 3D birth cohort

| Nearby CpG site | Chromosomic position (Hg19) | Beta value | SD | P value |
| --- | --- | --- | --- | --- |
| **cg22593959** | **chr7:121 184 995** | **-3.8** | **4.1** | **0.36** |
|  | chr7:121 184 789 | 5.1 | 16.5 | 0.76 |
|  | chr1:36 042 880 | -3,7 | 3.8 | 0.34 |
|  | chr1:36 042 902 | -2.8 | 3.3 | 0.39 |
|  | chr1:36 042 905 - 36 042 936 | -1.4 | 2.9 | 0.64 |
|  | chr1:36 042 945 | -5.1 | 2.9 | 0.08 |
|  | chr1:36 042 952- 36 042 987 | -2.3 | 3.4 | 0.50 |
|  | chr1:36 042 997 | -0.1 | 2.9 | 0.97 |
|  | chr1:36 043 003 | -0.59 | 2.6 | 0.82 |
|  | chr1:36 043 012 - 36 043 015 | -2.8 | 4.8 | 0.55 |
|  | chr1:36 043 020 | -0.2 | 2.6 | 0.94 |
|  | chr1:36 043 051 - 36 043 067 | -1.4 | 2.5 | 0.59 |
| **cg22436429** | **chr1:36 043 085** | **0.07** | **2.9** | **0.98** |
|  | chr1:36 043 112 - 36 043 132 | -1.7 | 2.5 | 0.49 |
|  | chr1:36 043 135 | -1.9 | 3.7 | 0.61 |
|  | chr1:36 043 164 - 36 043 173 | -2.3 | 2.9 | 0.42 |
|  | chr1:36 043 179 | -2.8 | 2.2 | 0.21 |
