# Supplementary figures and images for: Placental Epigenome-Wide Association Study Identified Loci Associated with Childhood Adiposity at 3 Years of Age
Source: Int J Mol Sci. 2020 Sep 29;21(19):7201. doi: 10.3390/ijms21197201 (PMC7582906; doi:10.3390/ijms21197201)

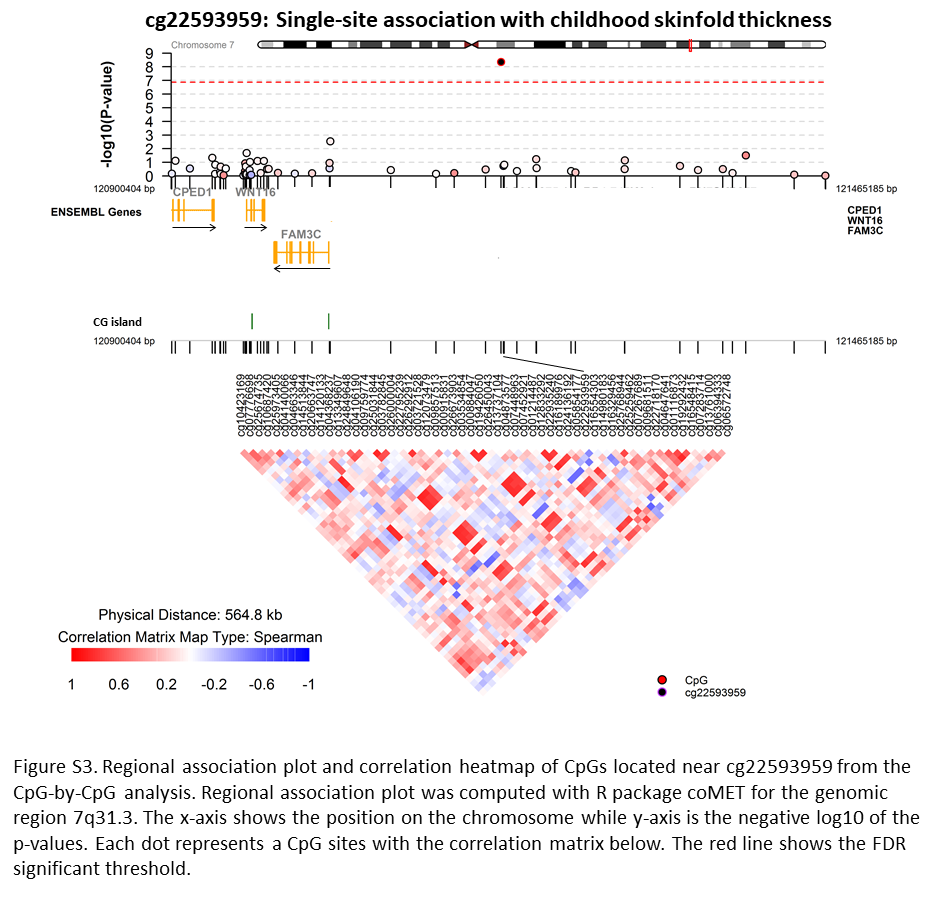

Supplement: Supplementary file 1 [file ijms-21-07201-s001.zip › Figure S1.PNG]

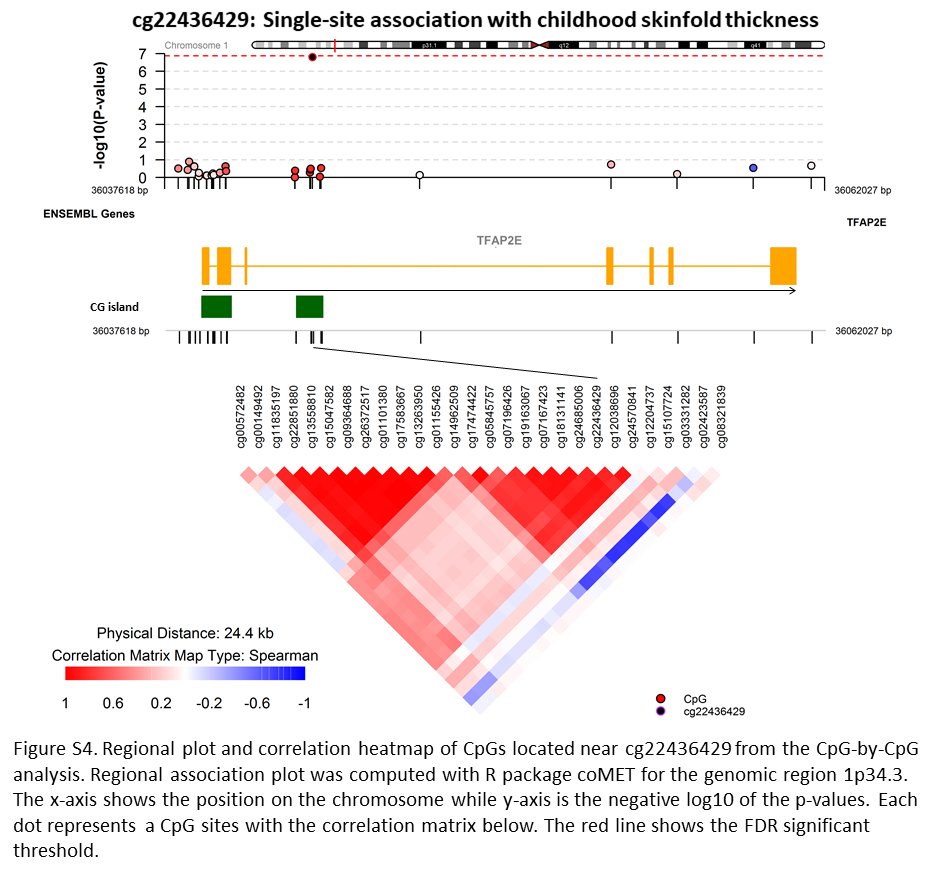

Supplement: Supplementary file 1 [file ijms-21-07201-s001.zip › Figure S2.png]

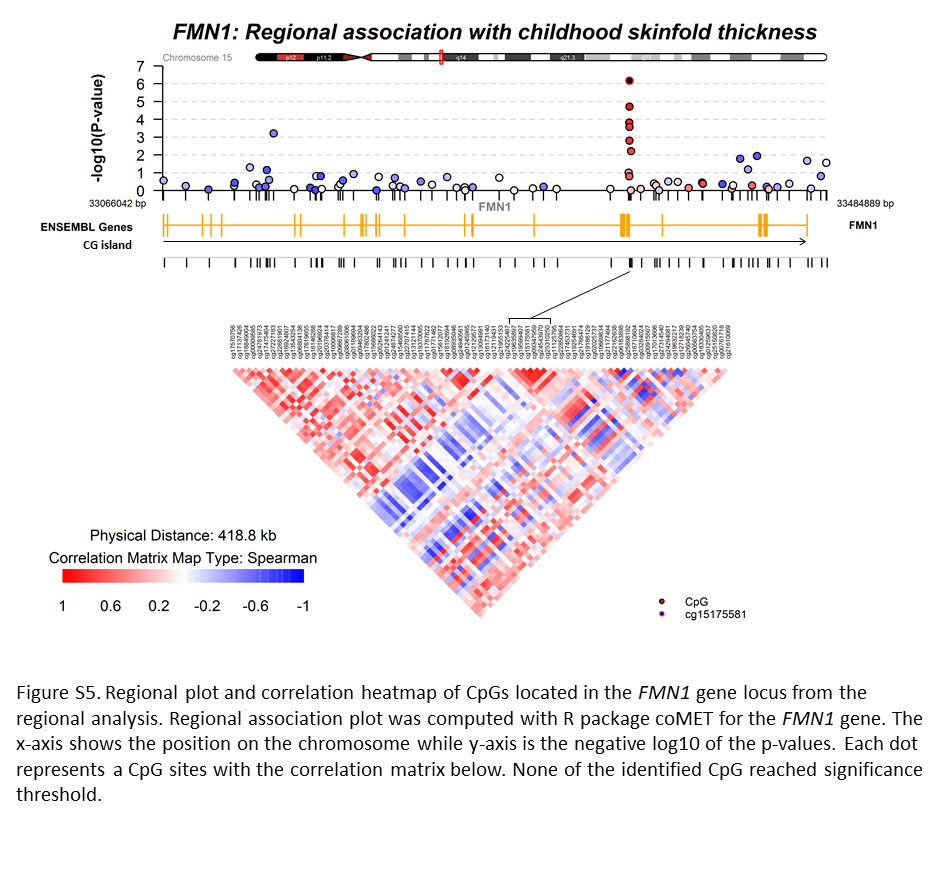

Supplement: Supplementary file 1 [file ijms-21-07201-s001.zip › Figure S3.png]

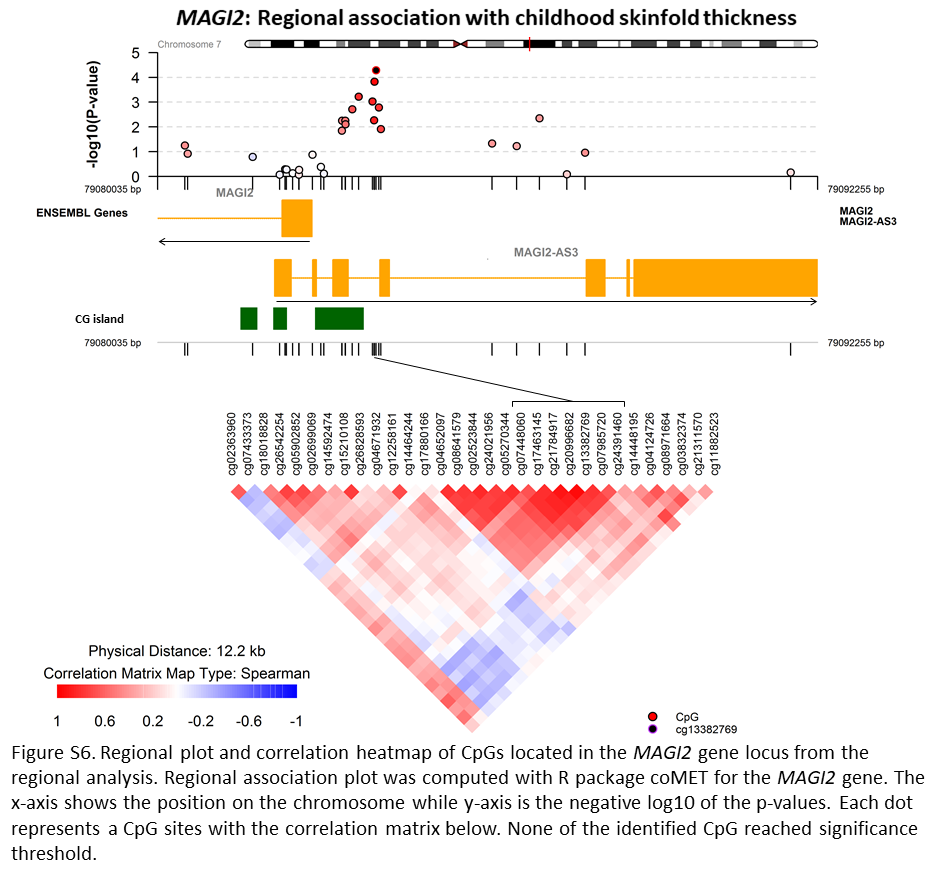

Supplement: Supplementary file 1 [file ijms-21-07201-s001.zip › Figure S4.png]

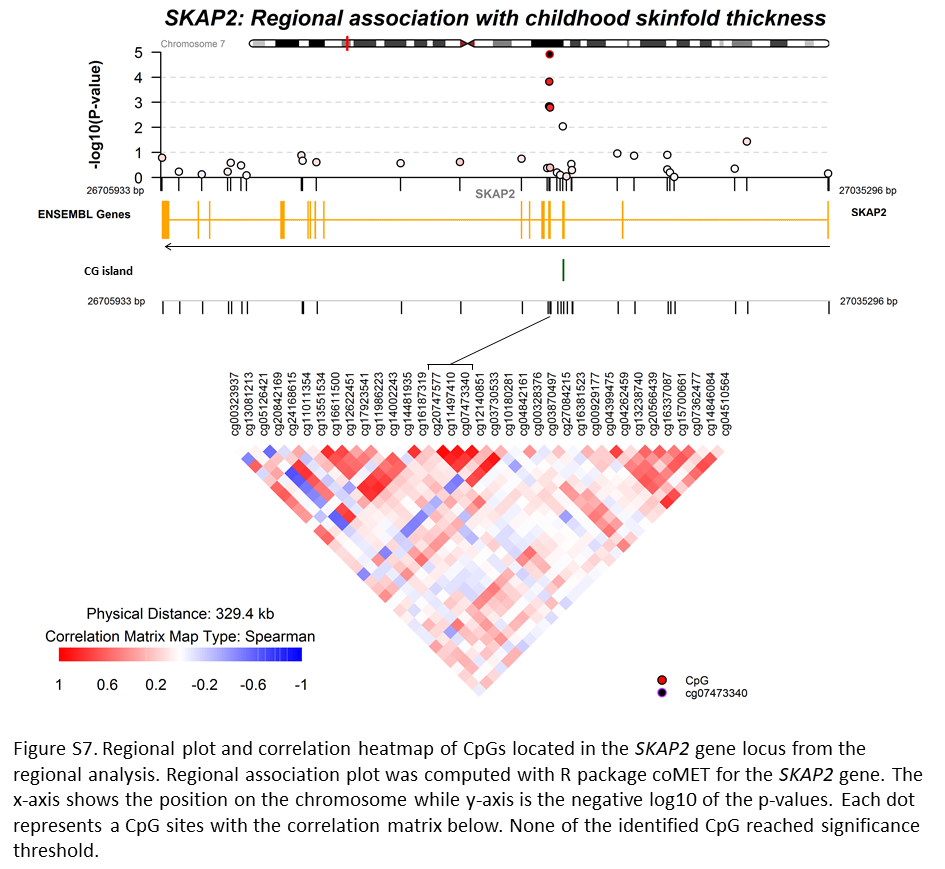

Supplement: Supplementary file 1 [file ijms-21-07201-s001.zip › Figure S5.png]

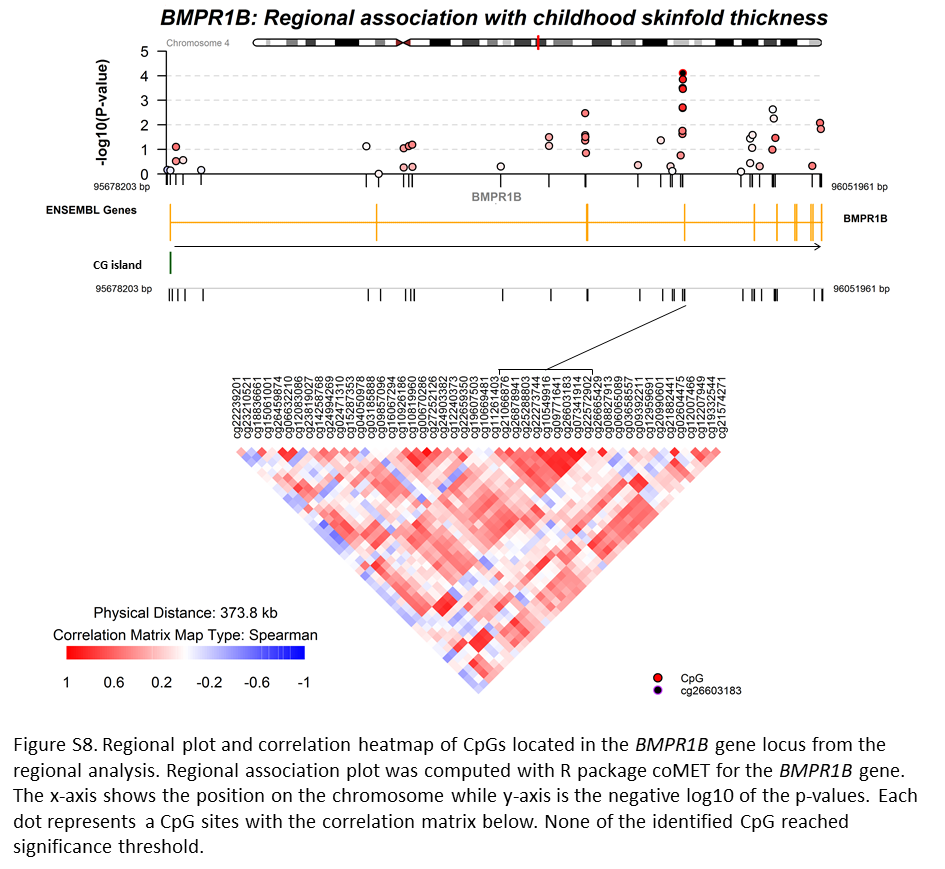

Supplement: Supplementary file 1 [file ijms-21-07201-s001.zip › Figure S6.png]

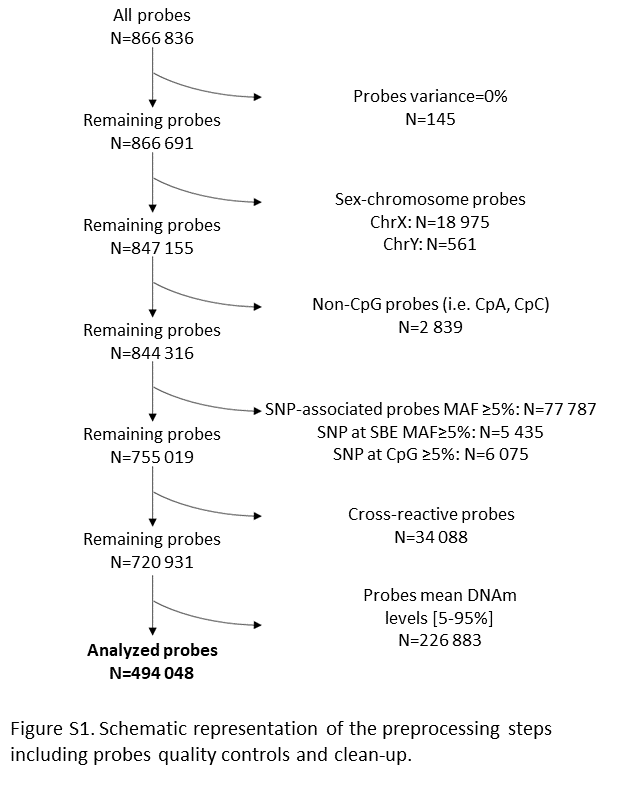

Supplement: Supplementary file 1 [file ijms-21-07201-s001.zip › Figure S7.png]

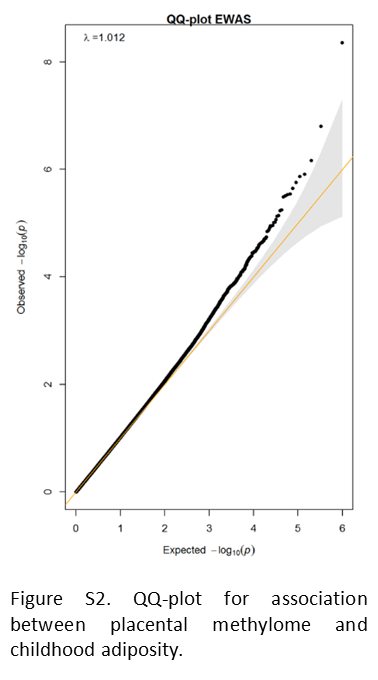

Supplement: Supplementary file 1 [file ijms-21-07201-s001.zip › Figure S8.png]
